# Effect, sensitivity, specificity and accuracy of ultrasonic assessment of axillary lymph node-negative breast cancer

**DOI:** 10.12669/pjms.39.5.7260

**Published:** 2023

**Authors:** Chun-Tian Hong, Yong-Hao Yan, Li-Yang Su, De-Bo Chen

**Affiliations:** 1 Chun-Tian Hong Department of Ultrasound, Quanzhou First Hospital Affiliated to Fujian Medical University, Fujian 362000, Quanzhou, P.R. China; 2 Yong-Hao Yan Department of Ultrasound, Quanzhou First Hospital Affiliated to Fujian Medical University, Fujian 362000, Quanzhou, P.R. China; 3 Li-Yang Su Department of Ultrasound, Quanzhou First Hospital Affiliated to Fujian Medical University, Fujian 362000, Quanzhou, P.R. China; 4 De-Bo Chen Department of Breast Surgery, Quanzhou First Hospital Affiliated to Fujian Medical University, Fujian 362000, Quanzhou, P.R. China

**Keywords:** Axillary Lymph Node (ALN), Breast cancer, Specificity, Color Doppler ultrasound, Sensitivity

## Abstract

**Objective:**

To investigate the diagnostic value of ultrasound for patients with axillary lymph node-negative breast cancer (ALNNBC).

**Methods:**

A retrospective analysis was performed on the clinical data of 204 breast cancer patients who were admitted by Quanzhou First Hospital Affiliated to Fujian Medical University between October 2020 and May 2022. According to the results of axillary lymph node (ALN) examination, the patients were assigned to a positive group(n=102) and a negative group(n=102). All patients underwent diagnosis with color Doppler ultrasound, with pathological diagnosis as the “gold standard” to determine the sensitivity and specificity of ultrasonic diagnosis. A receiver operating characteristic(ROC) curve was established to analyze the efficiency of ultrasonic diagnosis and compare the ultrasonographic features and flow grades between the two groups.

**Results:**

Differences were statistically significant between the two groups in ultrasonographic features of lesions(negative vs positive, all p<0.05), including morphological irregularity(59.8% vs 85.3%), spiky margins(19.6% vs 63.7%), posterior echo attenuation(19.6% vs 44.1%) and microcalcification(40.2% vs 55.89%). The negative group had a lower proportion of patients with grade 2-3 ultrasound blood flow when compared with the positive group(32.4% vs 56.86%), and the difference was statistically significant(p<0.05). Ultrasonic diagnosis of ALNNBC had a sensitivity of 88.24%(90/102), a specificity of 92.16%(94/102), a coincidence rate of 90.20% (184/204), a 95% CI of 0.845-0.928, and an AUC of 0.879.

**Conclusions:**

Ultrasonic diagnosis of ALNNBC is relatively efficient as ultrasonographic features and ultrasound blood flow signals can provide a scientific basis for the diagnosis of ALNNBC.

## INTRODUCTION

Breast cancer is one of the major malignancies that threaten women’s health in China. Clinical treatment varies depending on the patient’s condition. For axillary lymph node-negative breast cancer (ALNNBC), radical mastectomy remains the mainstay of treatment; in contrast, axillary lymph node dissection is required in the case of axillary lymph node-positive breast cancer.^1^-^2^ Clinically, there is no distinct difference between axillary lymph node (ALN)-negative and positive breast cancer, which share close similarities in terms of diagnostic imaging findings. Therefore, operation and pathology are currently regarded as the “gold standard” for the diagnosis of ALNNBC.

However, pathological examinations may cause damage to healthy tissues in the process of tissue sampling, an indispensable invasive procedure that limits the extensive use of pathological examinations in clinical practice.^3^ With continuous advances in the clinical use of color Doppler ultrasound, two- and three-dimensional (2D and 3D) ultrasound featuring easy-to-use, economical and high-resolution properties are complementary to conventional low-resolution ultrasound systems. In addition, color ultrasound enables a detailed probe into tissue blood flow, which substantially improves the efficiency in the diagnosis of ALNNBC.^4^-^5^ On this basis, the present study presents an analysis of the diagnostic efficiency of color ultrasound in patients with ALNNBC.

## METHODS

A retrospective analysis was performed on the clinical data of 204 breast cancer patients who were admitted by Quanzhou First Hospital Affiliated to Fujian Medical University between October 2020 and May 2022. Patient data including demographic data, diagnosis of breast carcinoma, were retrieved from electronic medical record systems.The patients were divided into a positive group(n =102, where axillary lymph node(ALN) was positive for breast carcinoma) and a negative group(n =102, where ALN was negative for breast carcinoma) by the results of ALN examination. All patients were diagnosed with infiltrating breast cancer.

### Ethical Approal

The study was approved by the Institutional Ethics Committee of Quanzhou First Hospital Affiliated to Fujian Medical University (No.:2018-109; October 19, 2018), and written informed consent was obtained from all participants.

### Inclusion criteria:

- Patients who met to the diagnostic criteria for breast cancer;^6^
- Patients received radical mastectomy at Quanzhou First Hospital Affiliated to Fujian Medical University and had complete pathology reports;
- Patients underwent ALN examination, of which the report remained intact;
- Patients had no history of any breast cancer surgery.

### Exclusion criteria:

- Patients had the nature of ALNs determined before ultrasonography;
- Patients had lesions in both breasts;
- Patients presented with uncontrolled bleeding;
- Patients was diagnosed with acute/chronic primary breast infection;
- Patients suffered from any severe diseases of the blood/endocrine system.

### Methods

All patients received color Doppler ultrasound examination (Philips Healthcare, Seattle, WA, United States, IU 22, IU Elite). After preparation a L12-5 high frequency linear array probe, a 7.5-MHz probe was used for general scanning in the bilateral breasts and armpits, while intralesional echoes were a C5-1 low frequency convex array probe. A 5-MHz probe was inserted to measure the dimensions and depth of the tumor, during which the patient was placed in a given position to fully expose her bilateral breasts and armpits for a detailed examination of each breast, with the nipple at the center and the probe being suspended next to the nipple without applying pressure on the site. The beam was steered in a slanting direction and introduced into below the nipple.

To avoid external interference, radial scanning was applied. Following that, longitudinal and transverse scanning were conducted multiple times, with the former from the anterior axillary line to the medial line and ending at the sternal border and the latter from the second intercostal space down to the sixth intercostal space. Color Doppler flow imaging (CDFI) was performed in parallel with scanning to visualize tissue blood flow and measure the resistance index (RI). Routine aspiration biopsy was employed under guidance of ultrasound, with the optimum site of aspiration of the shortest distance to the tumor.

After routine sterilization and local anesthesia, a fine needle was inserted through the skin into the lesion to sample tissue from the tumor. Biopsy was conducted after preparation of pathological sections made of the collected sample. Ultrasound scanning data of all patients were coded randomly and assessed by two professionals from Quanzhou First Hospital Affiliated to Fujian Medical University to reach a diagnosis through blind analysis and consultation. Ultrasound scanning methods and diagnostic criteria for breast cancer and ALN were subject to Ultrasonographic Diagnosis in Obstetrics and Gynecology.^7^

### Outcome measures:

*Ultrasonographic features:* Intergroup comparison of ultrasonographic features, including the shape, margins, posterior echoes and degree of calcification of lesions. *Grading of ultrasound blood flow:* Intergroup comparison of blood flow signals by Adler semiquantitative grading of all lesions which classifies intralesional blood flow into four grades^8^: grade 0: definite absence of blood flow signals in the lesion; Grade-1: one or two dotty or rod-like blood flow signals in a section; Grade-2: three or four dotty or rod-like blood flow signals, or a long vessel signal in a section; Grade-3: ≥5 dotty or rod-like blood flow signals ≥2 long vessel signals in a section.

*Ultrasound blood flow parameters:* Intergroup comparison of patients with lower RI (<0.7) and artery peak systolic velocity (PSV, <30 cm/s) and higher RI (≥0.7) and PSV (≥30 cm/s).

*Diagnostic efficiency of ultrasound in patients with ALNNBC:* With the definitive pathological diagnosis based on clinical data as the “gold standard”, the sensitivity and specificity of ultrasonic diagnosis of ALNNBC were calculated, and a receiver operating characteristic (ROC) curve was plotted to analyze the efficiency of ultrasonic diagnosis of ALNNBC. All patients was completed by the same group of surgeons.

### Statistical analysis

EpiData 3.1 was used for data entry and preprocessing and SPSS 22.0 for statistical analysis. Comparison of continuous data (age, disease course, BMI) under normal distribution curves and with equal variances was examined by the t-test. Comparison of unordered categorical data (clinical manifestations, ultrasonographic features, ultrasound blood flow parameters, cancer staging) was examined by the χ^2^ test. Blood flow grading was subject to the rank sum test. The efficiency of ultrasonic diagnosis of ALNNBC was evaluated by ROC curve analysis, and the area under the curve (AUC) was calculated. P<0.05 indicated a difference of statistical significance.

## RESULTS

There were no statistically significant differences between the two groups in age (t= 0.085), disease course(t=0.240), BMI(t=0.070), clinical manifestations(χ^2^ =0.040), pathological staging (χ^2^ =0.111) (All p>0.05). Table-I. Differences were statistically significant between the two groups in ultrasonographic features of lesions (negative vs positive, all P <0.05), including morphological irregularity (59.8% vs 85.3%), spiky margins (19.6% vs 63.7%), posterior echo attenuation (19.6% vs 44.1%) and microcalcification (40.2% vs 55.89%). Table-II.

**Table-I:** Intergroup comparison of baseline characteristics-1.

Group	n	Age Group (yrs)		BMI (kg/m²)	Disease course (mths)		
Negative Group	102	46.62±3.57		24.53±3.85	15.86±0.53		
Positive Group	102	46.55±3.69		24.59±3.76	15.82±0.59		
x^2^/t		0.085		0.070	0.240		
P		0.932		0.945	0.811		

*Group*	*n*	*Clinical manifestations*			*Pathological staging*		

		*Nipple discharge*	*Distending breast pain*	*Abnormalities of areola*	*Stage 1*	*Stage 2*	*Stage 3*

Negative Group	102	22(21.6)	17(16.7)	14(13.7)	15(14.7)	68(66.7)	19(18.6)
Positive Group	102	23(22.6)	19(18.6)	16(15.7)	13(12.8)	65(63.7)	24(23.5)
x^2^/t		0.040			0.111		
P		0.842			0.946		

**Table-II:** Intergroup comparison of ultrasonographic features (n, (%)2.

Group	n	Morphological characteristics			Margins		Posterior echoes		Microcalcification

		Regular	Irregular	Spiky	Not spiky	Attenuated	Not attenuated	Yes	No
Negative Group	102	41(40.2)	61(59.8)	20(19.6)	82(80.4)	20(19.6)	88(86.3)	41(40.2)	61(59.8)
Positive Group	102	15(14.7)	87(85.3)	65(63.7)	37(36.3)	45(44.1)	57(55.9)	57(55.89)	45(44.12)
x^2^		6.658			7.985		7.985		5.027
P		0.006			<0.001		<0.001		0.010

**Fig.1:**
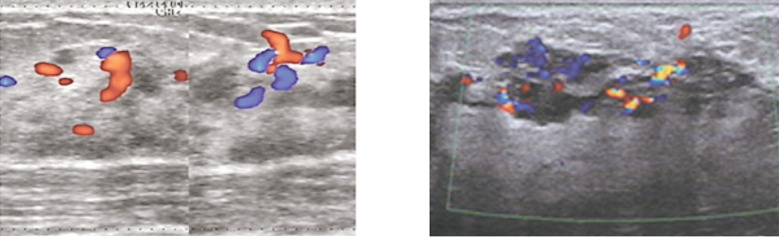
Typical ultrasound blood flows Left: early-stage infiltrating breast cancer, ALN-negative, 30-year-old, with a disease course of 3 months, grade-2 flow signals primarily distributed in the tissue surrounding the tumor and the intralesional streak-like hyperechoic area. Right: mid- to late-stage infiltrating breast cancer, ALN-positive, 36-year-old, with a disease course of 23 months, grade-3 flow signals mainly distributed in the intralesional solid hypoechoic area.

The negative group had a higher proportion of patients with grade 2-3 ultrasound blood flow when compared with the positive group (32.4% vs 56.86%), and the difference was statistically significant (p<0.05). Table-III. Typical ultrasound blood flows are as shown in Fig.1.

**Table-III:** Intergroup comparison of patients with different blood flow grades [n, (%)]3.

Group	n	Grade 0	Grade 1	Grade 2	Grade 3
Negative Group	102	4(3.9)	65(63.7)	26(24.5)	7(6.9)
Positive Group	102	1(0.1)	43(42.2)	35(34.3)	23(22.5)
Z		18.135			
P		<0.001			

The negative group had lower proportions of patients with an RI value <0.7 (90.2% vs 33.3%) and a PSV value <30 cm/s (100.0% vs 58.8%) as compared with the positive group , and the differences were statistically significant (p<0.05, respectively). Table-IV. According to the diagnostic data, ultrasonic diagnosis of ALNNBC had a sensitivity of 88.24% (90/102), a specificity of 92.16%(94/102) and a diagnostic accuracy of 90.20%(184/204), with the 95% confidence interval (95% CI) of 0.845 to 0.928 and the AUC of 0.879. Table-V.

**Table-IV:** Intergroup comparison of ultrasound blood flow parameters [n, (%)]4.

Group	n	RI		PSV	

		<0.7	≥0.7	<30 cm/s	≥30 cm/s
Negative Group	102	92(90.2)	10(9.8)	102(100.0)	0(0.0)
Positive Group	102	34(33.3)	68(66.7)	60(58.8)	42(41.2)
x^2^		7.899		6.925	
P		0.001		0.006	

**Table-V:** Results of ultrasonic diagnosis of ALNNBC5.

Diagnostic Method		Gold Standard		Total

		Positive	Negative	
Prenatal ultrasonography	Positive	90	8	98
	Negative	12	94	106
	Total	102	102	204

## DISCUSSION

The results of this study suggested that ultrasonographic features of lesions were less distinct in ALNNBC patients when compared with those with ALNPBC, including morphological irregularity (59.8% vs 85.3%), spiky margins (19.6% vs 63.7%), posterior echo attenuation (19.6% vs 44.1%) and microcalcification (40.2% vs 55.89%). This is consistent with the study results in Lin X et al.^9^, that is, ALNPBC is more likely to yield abnormal ultrasonographic results. In addition, it was shown that grade 2-3 ultrasound blood flow occurred less frequently in patients with ALNNBC than in those with ALNPBC (32.4% vs 56.86%); patients with ALNNBC were also more likely to have an RI <0.7 (90.2% vs 33.3%) and a PSV <30 cm/s (100.0% vs 58.8%) when compared with the ALN-positive cases.

These findings are in agreement with the study by Litvinova SP et al.,^10^ which demonstrates a greater intralesional blood flow in patients with ALNPBC. All these suggest that ultrasonography is useful in revealing the nature of ALNs and differentiating the margins, shape, posterior echoes, degree of calcification and blood flow of lesions in breast cancer patients, which thereby provides a scientific and accurate reference and basis for clinical diagnosis and treatment. Differences in ultrasonographic features are probably brought by the infiltrative growth and spread of breast cancer lesions to their peripheral tissue.

Early breast cancer has no noticeable signs or symptoms, and patients may develop lumps in the breast(s) and experience abnormalities of the breast(s) and skin as the disease progresses. Mid- to late-stage breast cancer has a range of symptoms that occur in parallel with cancer metastasis. This multiple malignancy is viewed as a grave threat to women’s health.^11^-^13^ In most cases, breast cancer patients can achieve a good prognosis as long as they receive symptomatic treatment in time.

However, treatment outcomes are highly dependent on early clinical diagnosis and choice of treatment according to the specific type of each lesion, which should be prioritized to improve the therapeutic effect and prognosis in patients with breast cancer.^14^-^16^ Clinically, breast cancer patients are provided with different treatment regimens according to their own condition and the severity of breast cancer. For ALNNBC at low risk of distant metastasis, the modified radical mastectomy is the mainstay of treatment, while axillary lymph node dissection is recommended to patients with ALN-positive breast cancer (ALNPBC).^17^

Therefore, it is essential to timely generate an accurate differential diagnosis between ALN-negative and -positive breast cancer in clinical settings to improve the therapeutic effect of subsequent treatment regimens.^18^ Ultrasound is widely used in the diagnosis of many diseases as a noninvasive technique characterized by its short wavelength, desired directionality, and the penetration depth of ultrasonic waves into opaque materials. Therefore, medical practitioners may establish a qualitative diagnosis of ALNs in breast cancer based on ultrasonographic findings to provide an important reference and basis for clinical diagnosis and treatment.^19^-^21^ On this basis, the present study presents an analysis of the diagnostic efficiency of color ultrasound in patients with ALNNBC.

Intralesional stromal reactions and peritumoral trabecular structures are gradually drawn towards the tumor; patients with ALNPBC often exhibit a greater degree of involvement of perilesional healthy tissue, which explains why ALNPBC cases usually have lesions characterized by morphological irregularity, spiky margins and substantial attenuation of posterior echoes.^22^

### Limitations

It includes a small sample size. In addition, we only analyzed and discussed the cases included in our hospital, which may not be representative enough. We look forward to a multi-center study in the future to reach more comprehensive conclusions.

## CONCLUSION

Ultrasonic diagnosis of ALNNBC is relatively efficient as the ultrasonographic features and blood flow signals offer a scientific basis for the diagnosis of ALNNBC. To improve the diagnostic accuracy, clinicians should carefully evaluate the ultrasonographic features of each suspected ALNNBC case, including margins, shape, posterior echoes, degree of calcification, and blood flow grading, as well as RI and PSV values.

### Authors’ Contributions:

**CTH** and **YHY:** Designed this study, prepared this manuscript, are responsible and accountable for the accuracy and integrity of the work.

**LYS:** Collected and analyzed clinical data.

**DBC:** Significantly participated in acquisition, analysis, and draft the manuscript. All authors read and approved the final manuscript.
